# Structural white matter characteristics for working memory and switching/inhibition in children with reading difficulties: The role of the left superior longitudinal fasciculus

**DOI:** 10.1162/netn_a_00257

**Published:** 2022-07-01

**Authors:** Rola Farah, Noam Glukhovsky, Keri Rosch, Tzipi Horowitz-Kraus

**Affiliations:** Educational Neuroimaging Group, Faculty of Education in Science and Technology, Faculty of Biomedical Engineering, Technion – Israel Institute of Technology, Haifa, Israel; Kennedy Krieger Institute, Baltimore, MD, USA; Department of Psychiatry and Behavioral Sciences, Johns Hopkins University School of Medicine, Baltimore, MD, USA

**Keywords:** Children, Reading difficulties, Executive functions, Fractional anisotropy, White Matter

## Abstract

Reading difficulties (RDs) are characterized by slow and inaccurate reading as well as additional challenges in cognitive control (i.e., executive functions, especially in working memory, inhibition, and visual attention). Despite evidence demonstrating differences in these readers’ language and visual processing abilities, white matter differences associated with executive functions (EFs) difficulties in children with RDs are scarce. Structural correlates for reading and EFs in 8- to 12-year-old children with RDs versus typical readers (TRs) were examined using diffusion tensor imaging (DTI) data. Results suggest that children with RDs showed significantly lower reading and EF abilities versus TRs. Lower fractional anisotropy (FA) in left temporo-parietal tracts was found in children with RDs, who also showed positive correlations between reading and working memory and switching/inhibition scores and FA in the left superior longitudinal fasciculus (SLF). FA in the left SLF predicted working memory performance mediated by reading ability in children with RDs but not TRs. Our findings support alterations in white matter tracts related to working memory, switching/inhibition, and overall EF challenges in children with RDs and the linkage between working memory difficulties and FA alterations in the left SLF in children with RDs via reading.

## Author Summary

Our findings support alterations in white matter tracts related to working memory, a component of executive functions, and reading in children with reading difficulties as well as the differences in association between white matter alterations in tracts associated with reading deficiency and working memory, switching/inhibition, and overall executive function challenges in children with reading difficulties.

## INTRODUCTION

### Reading Difficulty: Definition and Characteristics

Reading difficulty (RD) is defined as a specific reading disorder that affects individuals with average and above-average intelligence; cannot be attributed to environmental factors or neurological, psychiatric conditions, or brain damage; and is prevalent in about 10% of children in the United States (Lyon et al., 2003). The prevailing theory for the cause of RD is the phonological processing deficit, which stands for impairment in awareness of the spoken sounds in language and mentally mapping letters to representations of the corresponding speech sounds (phonemes) (Snowling, 1995). Moreover, several studies have recently pointed to additional challenges in cognitive control, or executive functions (EFs), in this group of readers (Horowitz-Kraus & Breznitz, 2014; Levinson et al., 2018; Meiri et al., 2019; Mercedes & Cutting, 2020; Pennington et al., 1993; Wang et al., 2012). More specifically, challenges in working memory (Fostick & Revah, 2018; Ram-Tsur et al., 2008; Smith-Spark & Fisk, 2007; Zhao et al., 2015), switching/inhibition (Levinson et al., 2018), and visual attention (Facoetti & Molteni, 2001) were reported. These findings are supported by the extension of the Simple View of Reading model (Cutting et al., 2015), suggesting that in addition to linguistic (phonological) and decoding/reading abilities, EFs play a central role in the reading comprehension process. More specifically, working memory was found to affect the linguistic and word decoding domains (Cirino et al., 2019; Kim, 2020; Spencer et al., 2020; Taboada Barber et al., 2021) with additional work supporting the role of switching/inhibition in word decoding as well (Spencer et al., 2020).

### Executive Functions: Definition and Relation to Reading Ability

EF is an umbrella term describing a set of high-order cognitive abilities that control and regulate functions and behaviors (Anderson, 2002). Basic EFs are involved in cognitive processes such as flexibility, working memory, attention control, and cognitive inhibition (Diamond, 2013; Lehto et al., 2003; Miyake et al., 2000). Studies have shown that children with RDs have deficits in working memory tasks in both verbal and visual domains and impairments in inhibition and shifting (Horowitz-Kraus, 2014; Reiter et al., 2005; Varvara et al., 2014). Reports suggest that the challenges in EFs in children with RDs continue into adulthood (Brosnan et al., 2002; Smith-Spark et al., 2016), interfering with their everyday routine and reading (Smith-Spark et al., 2016). Mechanistically, the ability to read newly encountered words relies on the ability to decode letters visually (i.e., utilizing visual attention abilities) to their corresponding sounds, maintain them in working memory, assemble them into a word, and match the semantic meaning to it in an automatic manner (see also Horowitz-Kraus, 2016). Switching/shifting between decoding and word recognition is also essential for fluent reading and reading comprehension (Spencer et al., 2020), which may explain how difficulties in these EFs contribute to reading challenges in children with RDs.

### Neurobiological Correlates of Reading and EF Dysfunction in Individuals With RDs

It is traditionally suggested that the neural reading network consists of three discrete left hemisphere regions: the temporo-parietal, inferior-frontal (inferior-frontal gyrus), and occipito-temporal (fusiform gyrus, aka the Visual Word Form Area) cortices and is associated with word recognition (Martin et al., 2015; Norton et al., 2015). The temporo-parietal region is related to language and phonological processing and comprehension, whereas the inferior-frontal regions are related to production and semantic processing (Dehaene, 2009). Interestingly enough, recent studies have pointed at the participation of frontal cortices related to EFs in reading: the dorsal-anterior cingulate cortex and the dorsolateral prefrontal cortex related to error monitoring and working memory, respectively, where greater activation was related to increased reading abilities (Buchweitz et al., 2019). This increased activation was extended to EF networks related to these regions (i.e., cingulo-opercular and fronto-parietal), which showed increased networks connectivity related to increased reading performance (Horowitz-Kraus & Holland, 2015; Patael et al., 2018; Turkeltaub et al., 2003). It was suggested that an engagement of frontal cortices and networks associated with EFs during reading was related to a compensatory mechanism for these readers (Horowitz-Kraus, 2014; Horowitz-Kraus et al., 2015a, 2015b; Horowitz-Kraus & Holland, 2015; Horowitz-Kraus et al., 2014).

Traditionally, the left hemisphere played a central role in intact reading abilities. Previous research suggested that typical reading (TR) involves activation of the aforementioned regions, mainly in the left hemisphere (Backes et al., 2002; Horowitz-Kraus et al., 2013; Shaywitz et al., 2002; Silani et al., 2005; Simos et al., 2002), whereas individuals with RDs showed activation in the right homologous regions (Backes et al., 2002; Shaywitz et al., 2002; Simos et al., 2002; Waldie et al., 2013). Additionally, an altered activation in the left hemisphere has also been consistently reported in individuals with RDs. Specifically, decreased activation of the left inferior parietal, superior temporal, middle and inferior temporal, and fusiform regions in adults (Richlan et al., 2009; Turkeltaub et al., 2003), children (Richlan et al., 2011), and prereaders at risk for RDs (Vandermosten et al., 2016) were related to lower reading skills (Hoeft et al., 2007).

Studies using diffusion tensor imaging (DTI) provide complementary information to these functional neuroimaging studies. The “classical” reading tracts that connect the reading-related gray matter regions mentioned above include the arcuate fasciculus (AF)—with studies reporting reduced fractional anisotropy (FA) in the left AF—negatively correlated with single-word reading skills (Gullick & Booth, 2015) and the left superior longitudinal fasciculus (SLF)—with findings of positive correlation between FA in the SLF and reading scores (Carter et al., 2009; Klingberg et al., 2000). In addition to the AF and SLF, the left inferior longitudinal fasciculus (ILF) connecting the posterior inferior temporal gyrus to the anterior and medial temporal lobe areas, plays a crucial part in organizing visual stimuli about words according to their lexical meaning (Anwander et al., 2007; Cummine et al., 2015; Qi et al., 2015; Yeatman et al., 2012a, 2012b; Yeatman et al., 2013). This tract has also been strongly linked to language and reading (Hoeft et al., 2011; Myers et al., 2014; Saygin et al., 2013; Yeatman et al., 2012a, 2012b).

The AF and SLF have also been linked to cognitive abilities in healthy children and adolescents, including attention and spatial working memory (Urger et al., 2015; Vestergaard et al., 2011). Traditionally, the left SLF had been implicated in working memory abilities (Vestergaard et al., 2011), with reported associations between spatial working memory and FA in the left SLF in healthy children 7–13 years old (Vestergaard et al., 2011). Similarly, FA in the left SLF was predictive of verbal working memory in healthy children 8–19 years old (østby et al., 2011) and healthy adults (Koshiyama et al., 2020). Taken together, the existing evidence shows altered structural connectivity related to reading in children with RDs. However, it is unclear whether there are shared white matter tracts for reading and EFs in general and working memory, visual attention and shifting/inhibition in particular in RDs.

Therefore, the current study aims to determine the structural differences in children with RDs versus TR related to their reading and EF abilities, focusing on tracts associated with both. Structural connectivity studies examining the neurobiological correlates for EFs have pointed to the left SLF, AF, and the ILF as the key structures associated with reading alterations (Farah et al., 2020; Muetzel et al., 2008; Pavuluri et al., 2009; Peters et al., 2014; Tamnes et al., 2012), and therefore these tracts were chosen in the current study. We hypothesized that children with RDs will demonstrate decreased reading and EF abilities (including working memory, switching/inhibition and visual attention) and alterations in tracts related to these abilities. We also hypothesized that variability in white matter microstructure pertaining to reading and EF abilities, more specifically the left SLF in children with RDs (per Wang et al., 2016), would predict working memory ability mediated by reading ability, indicating shared structural components. We suspect that better working memory ability in children with RDs will be influenced by better reading, echoing previous reports (Demoulin & Kolinsky, 2016).

## METHODS

### Participants

Children with RDs (*n* = 22, 10 females) and typical readers (TRs) (*n* = 24, 12 females) participated in the study, ages 8–12 years old with no significant mean age difference between the groups (*t*(44) = −1.183, *p* = 0.243). All participants were monolingual native English speakers with no neurological impairments or psychiatric history. Attention difficulties were exclusionary for this study and were determined using the Conners questionnaires (Goyette et al., 1978). Children in the RD group were diagnosed with RDs prior to study participation and demonstrated a standard score of −1 and below in at least two reading tasks from the reading tests in the “behavioral measures” list (following Kovelman et al., 2012; also see Behavioral Measures section). Both groups participated in the behavioral and neuroimaging sessions. Informed consents and assents were signed by parents and participants. The Institutional Review Board reviewed and approved the study in Cincinnati Children’s Hospital Medical Center, Ohio, USA.

### Behavioral Measures

#### General abilities.

General nonverbal abilities were measured using the Test of Nonverbal Intelligence (TONI) (Brown et al., 2010), and verbal abilities were measured using the Peabody Picture Vocabulary Test (PPVT) (Dunn & Dunn, 2007).

#### Reading measures.

Reading and reading-related abilities were assessed using the following reading assessments: (1) phonological processing, using the Elision subtest, Comprehensive Test of Phonological Processing (CTOPP; Wagner et al., 2013); (2) automatic word reading using the sight word efficiency (SWE), Test of Word Reading Efficiency (TOWRE; Torgesen et al., 1999); (3) automatic phonological decoding efficiency subtest (TOWRE; Torgesen et al., 1999); (4) nontimed word reading using the orthographical subtest (letter-word) (WJ III; Woodcock et al., 2001); and (5) nontimed decoding using the word-attack subtest (WJ III; Woodcock et al., 2001).

#### Executive functions measures.

EF abilities were assessed using the following age-normalized measures: (1) working memory (Digit Span, the Wechsler Intelligence Scale for Children, WISC; Wechsler, 2012); (2) switching/inhibition (DKEF Stroop, Color-Word Condition 3; Delis et al., 2001); (3) visual-spatial attention (Test of Everyday Attention for Children, TEA-Ch, Sky Search subtest; Manly et al., 1999); (4) overall EF skills (the Behavior Rating Inventory of Executive Function, BRIEF; Gioia et al., 2000).

### Behavioral Data Analysis

Independent samples *t* tests were used examine differences on the reading and EF tests between the two reading groups.

### Neuroimaging Data Acquisition and Procedures

Data were acquired using a 3-Tesla Philips Achieva scanner. A three-dimensional T1-weighted inversion recovery prepared anatomical whole-brain scan, MPRAGE sequence was acquired with the following parameters: TR/TE = 8.1/3.7 ms; matrix 256 × 224; 160 slices in the sagittal direction, 1.0-mm isotropic voxels; scan time: 5 m, 15 s. Diffusion data was acquired using a single-shot spin-echo, echo planar imaging with TR/TE = 6,652.446/82.60 ms, 61 gradient directions plus 7 b0 images, and b-value of 1,000 s/mm^2^, slice thickness = 2 mm, voxel size = 2 × 2 × 2 mm, field of view = 224 × 120 × 224 mm, for a total scan time of 7 m, 25 s.

Participants were acclimated and desensitized to prepare for comfortable testing inside the MRI Philips scanner (for the desensitization procedure, see Kraus & Horowitz-Kraus, 2022). Elastic straps were attached to either side of the head-coil apparatus, and a headband strap was put on the child’s forehead to control head motion. Headphones equipped with a built-in microphone were used to establish verbal communication between the child and the study coordinator, and video monitoring was used to assess the child’s state and movement inside the scanner.

### Neuroimaging Data Analyses

Preprocessing of the T1-weighted structural image included bias correction using the N4 algorithm (Tustison et al., 2010) as executed in the Advanced Normalization Tools toolbox. Removal of nonbrain tissue was completed using the Oxford Center for Functional MRI of the Brain brain extraction tool (Smith, 2002), after which the brain mask was applied to the original structural volume and bias correction using the N4 algorithm was repeated on the brain-extracted volume. Segmentation was performed using the FSL FIRST to produce a 3-class tissue (Zhang et al., 2001).

DTI data were processed using the Vistalab diffusion MRI software suite (Stanford Vision and Imaging Science and Technology) as part of the open-source mrDiffusion package: https://white.stanford.edu/software. DTI images were aligned to the motion-corrected mean of the nondiffusion-weighted (b = 0) images by using a rigid body algorithm. Following realignment, DTI images were then resampled to 2-mm isotropic voxels with eddy current and motion correction using a seventh-order b-spline algorithm based on statistical parameter mapping. Finally, the diffusion tensors were fitted to the resampled DTI data by using a least-squares fit and the RESTORE (robust estimation of tensors by outlier rejection) algorithm (Chang et al., 2005). The diffusion tensor model produces measures describing the diffusion characteristics of each voxel. Eigenvalues (λ_1_, λ_2_, λ_3_) from the diffusion tensor were used to compute FA (√(1/2)√((λ_1_ − λ_2_)2 + (λ_3_ − λ_2_)2 + (λ_3_ − λ_1_)2)/√(λ_12_ + λ_22_ + λ_32_) (Pierpaoli et al., 1996).

Using an in-house pipeline utilizing automatic fiber quantification (AFQ) software tool (Yeatman et al., 2012a, 2012b), the target white matter tracts in the brain of each participant were identified. Target white matter tracts included the AF, SLF, and ILF. Several processing steps were applied for each of the tracts including (1) whole-brain tractography, (2) region-of-interest (ROI)-based fiber tract segmentation and cleaning using a statistical outlier rejection algorithm, and (3) FA quantification. For tracking of target fasciculi, an initial seed point within the white matter mask was detected, and streamlines in both directions along the principal diffusion axes were traced. Tracing was terminated under two standard criteria: (1) if the FA at the current location was less than 0.2 and (2) if the minimum angle between the last path segment and next step direction is greater than 30 degrees (Yeatman et al., 2012a, 2012b). Each fiber tract was sampled to 99 equidistant nodes, and the spread of fibers at each node was represented as a three-dimensional Gaussian distribution. Fibers that were more than 5 standard deviations from the mean of the tract were removed. This procedure was repeated until no fiber outliers existed. Next, a quantification phase was conducted following the initial AFQ processes, where the diffusion properties were calculated by interpolating the FA values along the trajectory of the fiber group. Finally, mean and variance were calculated ‘within’ and ‘between’ groups for FA in each tract in the current analysis. The characteristics evaluated for each node and tract were later used to compare the groups.

### Diffusion Data Analysis: Group Comparison

#### Fractional anisotropy tract profile comparison between the groups.

For the statistical analyses of FA, cluster-based analyses were conducted for the 99 nodes between each tract’s defining ROIs (Yeatman et al., 2012a, 2012b). The analyses were performed using independent samples *t* tests comparing RD and TR groups.

To compare tract profiles between the RD and TR groups, multiple two-tailed *t* tests were conducted, and a permutation-based multiple comparisons correction (Nichols & Holmes, 2002) was used to calculate clusters with adjacent *t* tests with significant differences between the groups. Significance was corrected for multiple comparisons, and the corrected alpha was set to 0.05 (Nichols & Holmes, 2002; Yeatman et al., 2012a, 2012b). The clusters that were reported showed the following criteria: (1) all neighboring nodes were significantly different (*p* < 0.05, uncorrected) between the groups and (2) the cluster of significant values was larger than the critical cluster size generated by the permutation-based multiple comparisons correction (Nichols & Holmes, 2002; Yeatman et al., 2012a, 2012b).

### Correlations Between Diffusion and Behavioral Measures in the Reading and EF-Related Tracts

Using a Statistical Package for the Social Sciences (SPSS for Windows, version 24), correlation analyses were conducted between the average FA in clusters showing significant group differences and the participants’ (1) reading measure (TOWRE-SWE) and (2) EFs for each group separately. Normality was assessed based on Kolmogorov–Smirnoff (Corder & Foreman, 2009). Pearson correlation was reported when both correlation variables had normal distribution, and Spearman correlation was used for cases where the measures were not normally distributed in a specific group. Multiple correlations were corrected using a Bonferroni correction (*p* < 0.05). Fisher *z*-transformations assessed the significance of differences between the correlation coefficients measured in each group separately and the differences in correlations between the groups (Sheskin, 2004).

### Moderated Mediation Analysis

**Figure 1.**
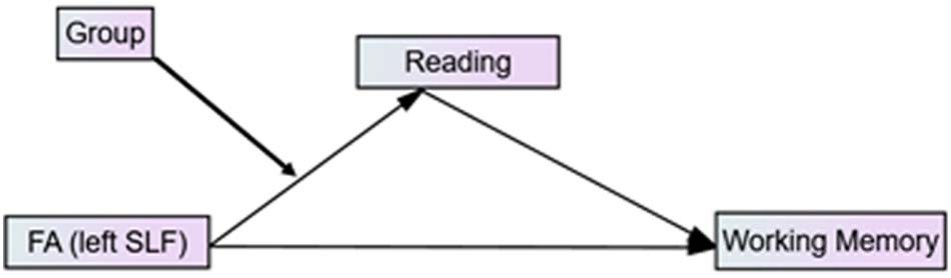
Conceptual moderated mediation model for the predicting role of white matter microstructure on working memory ability mediated by reading ability and moderated by group. Predictor: FA of the left SLF, Outcome: working memory, mediator: reading ability, moderator: reading group.

A moderated mediation analysis was conducted to test if the relationship between FA in the left SLF and working memory performance was mediated by reading ability and moderated by group (RD vs. TR) (Hayes, 2013). Therefore, our hypothetical model links FA in the left SLF to working memory via an indirect path that includes reading ability (as a mediator), as a function of group (moderator), resulting in a conditional indirect effect. Bootstrapping bias-corrected confidence intervals were used with 10,000 bootstrap samples to test the null hypothesis (i.e., the indirect effect of FA in the left SLF on working memory is not significant). When zero falls within the confidence intervals, the null hypothesis is accepted. PROCESS macro (version 3.4) for SPSS was used for data analysis (Hayes, 2013). The index of moderated mediation was used (Hayes, 2015), and bootstrapping bias-corrected intervals was used to test its significance. Figure 1 illustrates the second stage moderated mediation model to be tested.

## RESULTS

### Behavioral Measures

#### General abilities.

No significant differences between children with RDs and TRs were observed in general cognitive abilities and attention abilities (measured by the Conners). Decreased general language ability (as measured by the PPVT test) was found in children with RDs versus TRs (see Table 1; data was corrected for multiple comparisons using a Bonferroni correction *p* < 0.05).

**Table 1.** Baseline behavioral reading and executive functions scores for children with reading difficulties and typical readers

Cognitive ability			Children with RDs Mean (*SD*) (A)	TR Mean (*SD*) (B)	Contrast (direction of results)	*T* score
General ability	General verbal ability	Language ability (PPVT, standard score)	100.59 (8.87)	110.95 (12.79)	A < B	3.05*
	General nonverbal ability	Non-linguistic ability (TONI, percentile)	53.19 (22.69)	54.87 (21.77)	A < B	−0.25
Reading	Word-level reading	Word reading (TOWRE, scaled score)	79.41 (13.12)	105.96 (13.07)	A < B	6.87***
		Nonword reading (TOWRE, scaled score)	78.86 (11.13)	108.79 (11.57)	A < B	8.92***
		Word reading, nontimed (WJ, letter-word, standard score)	86.09 (12.61)	112 (9.98)	A < B	7.76***
	Phonological processing	Phonological processing (CTOPP, Ellison, scaled score)	7.27 (2.1)	11.83 (2.33)	A < B	6.95***
Executive functions	Working memory	WISC Digit Span (standard score)	8.77 (2.11)	9.96 (1.79)	A < B	2.03*
	Visual-Spatial/auditory Attention	Teach TEA-Ch Sky Search Attention Test (scaled score)	6.77 (2.62)	9.67 (3.52)	A < B	3.14**
	Switching/inhibition	DKEF Color-Word Condition 3 (standard score)	8.55 (3.02)	14.63 (3.52)	A < B	4.37***
	General EF score	BRIEF General Cognitive (parental reported) (*t* score)	68.57 (9.69)	42.54 (10.65)	A > B	3.1**

*Note*. PPVT, Peabody Picture Vocabulary Test; TONI, Test of Nonverbal Intelligence; TOWRE, Test of Word Reading Efficiency; WJ, Woodcock-Johnson; CTOPP, Comprehensive Test of Phonological Processing; DKEF, Delis-Kaplan Executive Function System; BRIEF, Behavior Rating Inventory of Executive Function; TEA-Ch, Test of Everyday Attention for Children. Results are presented as mean (standard deviation). *p < 0.05; **, *p* < 0.01; ***, *p* < 0.001. Note that a higher score in the BRIEF represents lower abilities.

#### Reading abilities.

Children with RDs demonstrated significantly decreased reading abilities versus TR (timed and nontimed word and nonword reading subtests, reading fluency subtests, and phonological processing tests). See Table 1 for these results.

#### Executive functions.

Children with RDs showed significantly decreased EF abilities in several subdomains: switching/inhibition (DKEF subtest), working memory (WISC Digit Span test), and overall EF skills (BRIEF), and visual attention (Sky Search visual attention test from the TEA-Ch battery). Note that for the BRIEF questionnaire, a higher score is related to a lower EF ability. See Table 1.

### Correlations Between Reading Abilities and Behavioral Executive Functions Across Both Groups

Overall, greater reading scores were associated with greater EF abilities across both groups. Specifically, significant correlations were found between automatic reading ability (TOWRE-SWE) and inhibition (*r* = 0.598, *p* < 0.001, measured by the Color-Word subtest, DKEF); working memory (*r* = 0.55, *p* < 0.001, measured by Digit Span test, WISC); General EF score (*r* = −0.5, *p* < 0.001, GEC test, BRIEF); and visual-spatial attention (*r* = 0.398, *p* = 0.006, Sky Search Attention test, TEA-Ch). Data were corrected for multiple comparisons using a Bonferroni correction at *p* < 0.05.

### Diffusion Tensor Imaging Results—Comparison of Fractional Anisotropy Values in Reading and EF-Related Tracts Between the Groups

**Figure 2.**
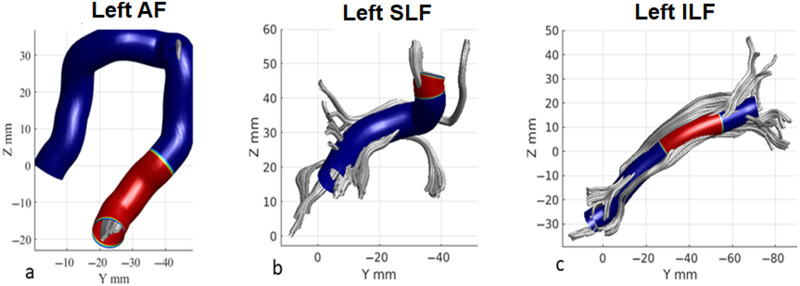
Location of significant node clusters with significant difference between children with reading difficulties and typical readers (red) in the left arcuate fasciculus (A), left superior longitudinal fasciculus (B) and left inferior longitudinal fasciculus (C).

Fractional Anisotropy: Overall, children with RDs showed significantly lower FA in the left AF, ILF, and SLF, compared to TRs. See Table 2 and Figure 2 for the number of significant nodes per tract and the comparisons between the groups.

**Table 2.** Node cluster-based *t* test analysis for white matter tract DTI measures in children with reading difficulties and typical readers

Tract	Number of nodes with significant (*p* < 0.05) difference between children with RDs and TRs (location of cluster by node number)	Contrast (direction of results)
Left AF	20 (79–99)	TR > RD
Left SLF	9 (91–99)	TR > RD
Left ILF	28 (26–53)	TR > RD

*Note*. Location of node clusters with a significant difference in fractional anisotropy values between children with reading difficulties (RDs) and typical readers (TRs). Results are shown as a total number of nodes with significant differences (specific location along the tract denoted by the node numbers). The contrast column shows the directionality between the groups in these clusters. AF, arcuate fasciculus; SLF, superior longitudinal fasciculus; ILF, inferior longitudinal fasciculus.

### Correlations of Reading Ability and Fractional Anisotropy—Correlations Between Reading (TOWRE-SWE) and Fractional Anisotropy Cluster Values

Higher reading ability was associated with higher FA values in the left SLF in children with RDs (*r* = 0.712, *p* < 0.001). No significant correlation was found between FA in the left SLF and reading in TRs (*r* = −0.132, *p* = 0.270). See Table 3.

**Table 3.** Correlations between reading, executive functions, and fractional anisotropy cluster values in children with reading difficulties and typical readers in temporo-parietal white matter tracts

	Behavioral ability	Behavioral Measure	Left AF *r* (*p*)	Left SLF *r* (*p*)	Left ILF *r* (*p*)
**Reading Difficulties**	Reading	TOWRE-SWE	−0.34 (0.06)	0.712 (**<0.001**)	0.04 (0.42)
	Switching/inhibition	DKEF Color-Word Condition 3	−0.36 (0.05)	0.31 (0.08)	0.32 (0.07)
	Working Memory	WISC Digit Span	−0.06 (0.39)	**0.68** (**0.002**)	−0.23 (0.15)
	General EF	BRIEF GEC	0.012 (0.47)	0.14 (0.28)	−0.19 (0.21)
	Visual-Spatial Attention	TEA-Ch Sky Search	−0.16 (0.24)	0.24 (0.14)	0.12 (0.29)
**Typical Readers**	Reading	TOWRE-SWE	0.02 (0.46)	−0.13 (0.27)	0.16 (0.23)
	Switching/inhibition	DKEF Color-Word Condition 3	−0.02 (0.46)	−0.3 (0.08)	0.14 (0.25)
	Working Memory	WISC Digit Span	−0.06 (0.39)	0.18 (0.19)	0.02 (0.46)
	General EF	BRIEF GEC	0.09 (0.33)	−0.16 (0.22)	0.36 (0.04)
	Visual-Spatial Attention	TEA-Ch Sky Search	0.21 (0.16)	0.29 (0.08)	0.32 (0.07)

*Note*. Reading, TOWRE sight word reading, inhibition – DKEF condition 3, working memory – WISC Digit Span, general EFs – BRIEF GEC, visual attention – TEA-Ch Sky Search. Results are presented as correlation coefficients (*p* value). AF, arcuate fasciculus; SLF, superior longitudinal fasciculus; ILF, inferior longitudinal fasciculus. Significant results are bolded. Note that a negative correlation coefficient for the correlation of FA with the BRIEF test was noted as a positive correlation with ability since lower BRIEF scores suggest less EF difficulties.

Children with RDs versus TRs: The correlation coefficients calculated in the two groups differed significantly (Fisher’s *z* = 3.43, *p* < 0.001); that is, children with RDs showed a greater positive correlation between FA in the left SLF and reading compared to TRs. See Table 4.

**Table 4.** Fisher *z*-transformations of correlation coefficients in fractional anisotropy between children with reading difficulties (r1) and typical readers (r2)

Behavioral ability/tract	Behavioral Measure	Left AF *z* (*p*)	Left SLF *z* (*p*)	Left ILF *z* (*p*)
Reading	TOWRE-SWE	−1.2 (0.11)	3.23 **(0.001)**	−0.37 (0.35)
Working Memory	WISC Digit Span	−0.00 (0.5)	2.14 **(0.016)**	−0.8 (0.21)
Switching/inhibition	DKEF Color-Word Condition 3	−1.11 (0.13)	1.99 **(0.02)**	0.61 (0.27)
Visual attention	TEA-Ch Sky Search	−1.19 (0.12)	−0.19 (0.42)	−0.64 (0.26)
General EF	BRIEF GEC	−0.24 (0.4)	0.96 (0.17)	−1.79 **(0.037)**

*Note*. Reading, Test of word reading efficiency (TOWRE), working memory – Wechsler Intelligence Scale for Children (WISC), Digit Span task. Results are presented as z-score (*p* value). AF, arcuate fasciculus; SLF, superior longitudinal fasciculus; ILF, inferior longitudinal fasciculus. Significant results surviving multiple comparisons are bolded.

### Correlations Between Executive Functions and Fractional Anisotropy—Correlations Between Executive Functions Measures and Fractional Anisotropy Cluster Values

A significant positive correlation was found between working memory and FA in the left SLF cluster in children with RDs (*r* = 0.612, *p* = 0.005) but not in TRs (*r* = 0.184, *p* = 0.195). In the left ILF, a trend was observed; however, it did not reach significance following the control for false discovery rate. See Table 3.

For children with RDs versus TRs, A direct comparison of the correlation coefficient values between the groups using Fisher’s *z*-transformation was conducted. The results suggest that the magnitude of the correlation coefficient for children with RDs significantly exceeded that of TRs in working memory (left SLF cluster) and cognitive flexibility (left ILF cluster). See Table 4.

### Moderated Mediation Analysis

Table 5 summarizes the overall model (regression coefficients, standard errors, *t* value, and significance).

**Table 5.** Statistics of the moderated mediation model

	**b**	***SE***	***T***	***P***
**Outcome variable: Reading variable**				
Constant	49.18	5.23	9.38	>0.001
FA, left SLF (predictor)	298.71	140.04	2.04	0.042
Group (moderator)	29.83	3.26	9.12	>0.001
FA, left SLF × Group (interaction)	−176.89	84.88	−2.08	0.043
*F*(3, 42) = 29.40, *P* = 0.00, *R*^2^ = 0.67				
**Outcome variable: Working memory**				
Constant	4.42	1.24	3.56	0.0009
FA, left SLF (predictor)	4.05	5.94	0.68	0.498
Reading (predictor)	0.053	0.012	4.14	0.0002
*F*(2, 43) = 8.96, *P* = 0.0006, *R*^2^ = 0.29				

Higher FA in the left SLF was associated with higher reading score (b = 298.71, *p* < 0.05). Higher FA in the left SLF was not directly associated with higher working memory scores (direct effect; b = 4.05, *p* = 0.498). The group variable significantly moderated the relationship between SLF and reading (b = −176.89, *p* = 0.043). However, the interaction only estimated the effect of FA in left SLF on reading by group and did not quantify the relationship between the moderator and the indirect effect.

Therefore, a formal test of moderated mediation was conducted, given by the index of moderated mediation (Hayes, 2015). The indirect effect of FA in the left SLF on working memory through timed word reading was dependent on the group and proved significant, as the bootstrap confidence interval (CI) of the index of moderated mediation did not contain zero (index = −9.46, S.E. = 5.22; 95% CI −.02, −1.78).

Table 6 presents the conditional indirect effect at two values of the dichotomous moderator: zero (RD group) and 1 (TR group). The findings indicated that higher FA in the left SLF led to higher working memory scores through higher reading scores only in the RD group.

**Table 6.** Conditional indirect effects of FA on working memory through reading ability at values of the moderator (group)

Group	Effect	Boot *SE*	95% CI	Significance
0 (RD)	6.51	3.71	1.34, 15.76	Significant
1 (TR)	−2.94	3.07	−9.79, 2.401	Nonsignificant

## DISCUSSION

The goal of the current study was to determine the shared structural alterations associated with EFs and reading in children with RDs. Per our hypotheses, the results demonstrated that children with RDs showed lower reading and EF abilities associated with decreased FA in the left SLF and ILF. Furthermore, our results support previous studies pointing at reduced FA in the left AF and SLF (dorsal pathway, phonology, and working memory-related) and ILF (ventral pathway, semantic/orthography related) (Rimrodt et al., 2010; Steinbrink et al., 2008; Su et al., 2018; Vandermosten et al., 2012, 2015). Support for the inferiority of children with RDs utilizing their left hemisphere was also found in the current study: children with RDs have shown lower FA in left temporo-parietal regions compared to TR. However, those with RDs who read better utilized their left hemisphere more: a higher positive correlation of reading and EFs with FA in the left SLF and ILF. These results will be discussed in depth in the context of the “extended” Simple View of Reading model (Cutting et al., 2015), outlining the involvement of EFs, and especially working memory, and switching/inhibition in the reading process in these readers.

### The Relations Between Executive Functions and Reading in Children with RDs

Children with RDs have shown lower EF abilities correlated with their reading. This echoes previous studies that examined the role of EFs in children with RDs in general and in reading in particular (Brady et al., 1983; Jorm, 1979; Reiter et al., 2005; Varvara et al., 2014). Our study was built upon previous findings, which demonstrated that reading abilities in children with RDs are related to decreased EF ability compared to their TR counterparts (Bailey et al., 2018; Farah et al., 2019; Haft et al., 2019; Horowitz-Kraus et al., 2014; Mercedes & Cutting, 2020). The findings in the current study strengthen the “extended” Simple View of Reading model (Cutting et al., 2015), outlining the involvement of EFs in the reading process, especially the connection between working memory, switching/inhibition, and word decoding/recognition (Spencer et al., 2020). A recent study has demonstrated how an EF-based reading training had a positive effect on EFs, reading, and increased functional connectivity between neural circuits, supporting both reading and EF (fusiform gyrus and the dorsal part of the anterior cingulate cortex) in children with RDs (Horowitz-Kraus & Holland, 2015). This further supports the reliance of word reading on EFs in children with RDs and their close association to their reading difficulties. Future studies are warranted to examine the effect of an EF-based reading intervention on tracts related to reading and EFs in children with RDs and TRs.

### Reduced Engagement of the Left Hemisphere in Children With RDs

Our results suggest that children with RDs demonstrated lower FA in left temporo-parietal tracts compared to children with TR. In support of our results, the findings of alteration in structural connectivity in the corpus callosum, forceps major, and vertical occipital fasciculus in participants with RDs versus TRs were previously reported in Finnish as well as in Chinese speakers (specifically in the left inferior fronto-occipital fasciculus, cerebellar pathways, and thalamo-pontine tracts and the posterior isthmus and anterior splenium of the corpus callosum) (Sihvonen et al., 2021; Wang et al., 2019) . However, whereas these studies examined the correlations between these alterations with phonological processing (English) and Chinese characters and auditory processing in the Chinese cohort, our study extends these findings also to include the correlation between SLF and working memory and switching/inhibition abilities.

It is important to mention, though, that a recent activation likelihood estimation meta-analysis on adult and pediatric populations focusing on voxel-based analysis and employing more drastic corrections for multiple comparisons reported no reliable differences between children with RDs and TRs in FA (Moreau et al., 2018). In addition to the rigor and correction resulting from a whole-brain analysis, this study also included a wide range of age groups, which have variable reading abilities and disorders and might have diminished the difference between groups in that study and reported in the current one. Previous studies documented decreased activation/hypoactivation in left temporo-parietal and right prefrontal gray matter regions associated with the reading network in individuals with RDs (Hoeft et al., 2011; Waldie et al., 2013). In addition to brain activation, individuals of all ages with RDs also showed decreased gray matter volume and altered sulci patterns in left occipito-temporal and temporo-parietal brain regions compared with TRs (Hoeft et al., 2007; Im et al., 2016; Pernet et al., 2009; Richlan et al., 2009). These reported findings might be related to the reduced FA in the left hemisphere found in the current study in readers with RDs; however, this should be further examined. A joint functional MRI-DTI study is needed to verify this point.

Our results also show a higher positive correlation between reading, working memory, switching/inhibition, and FA in the left SLF in children with RDs compared to TRs. The correlation comparisons provide structural support to the previously suggested role of the left hemisphere in reading and EF abilities (Barbey et al., 2012; Gonzalez et al., 2014; Hunter & Sparrow, 2012; Illingworth & Bishop, 2009; Leonard & Eckert, 2008; Shaywitz et al., 1998). Our findings suggest that higher FA in left temporo-parietal tracts (ILF and SLF) among children with RDs is related to better reading and EF abilities in this population. These results are complemented by previous studies showing adjacent gray matter volume indices in the left occipito-temporal and temporo-parietal areas in children with RDs, which correlate positively with reading and reading-related skills (He et al., 2013; Kronbichler et al., 2008). The correlations found between the FA in the SLF and working memory scores is supported by other findings related to the role of the left inferior parietal lobule in working memory/verbal memory processing (e.g., Borst & Anderson, 2013). The researchers reported a correspondence between updating working memory and activation in the inferior parietal lobule. As the SLF interconnects the inferior parietal regions with the frontal lobe and subserves fronto-parietal network crucial for efficient working memory (and EFs), our results correspond with Borst and Anderson regarding the relations of the SLF to working memory and switching/inhibition abilities. Moreover, the connection between the ILF and EFs, echoes previous findings (Fjell et al., 2016) and can be explained by the anatomy of the ILF connecting the occipital and more anterior brain regions through the temporal lobe, all associated with parts of the reading network (Cohen & Dehaene, 2009; Dehaene, 2009) and EF networks (Dosenbach et al., 2008). It would be interesting to conduct a multimodal functional-structural MRI study to confirm the functional alterations in reading and EF networks with the structural data in children with RDs. Importantly, the lack of association between the selected white matter tracts and EFs and reading in TRs, may point at alternative network recruitment needed to excel in these abilities among this population. It might be that for these readers, word reading level is relatively automatic and therefore does not demand the recruitment of EFs. However, the utilization of EF and reading-related neural circuits might be needed for contextual reading and reading comprehension (Meri et al., 2020). This was also observed in Meri et al. (2020) in an fMRI-based study focusing on reading comprehension and EF networks in TRs and children with RDs.

### Mediating Effect of Reading on the Relation Between FA in the Left SLF and Working Memory in Children with RDs

The data of the current study provide support for poor reading ability as a common neuropsychological deficit that links FA in the left SLF and working memory ability among children with RDs. The results indicated that FA in the left SLF was related to reading ability, which in turn influenced working memory ability, and that this indirect effect was moderated by the reading group (RD vs. TR). More specifically, the indirect effect of FA in the left SLF on working memory through reading was significant only in children with RDs but not in the TR group. Per our hypothesis, better working memory ability in children with RDs was influenced by better reading, as learning to read might shape immediate memory (Demoulin & Kolinsky, 2016), also suggested by Nick Ellis about 30 years ago (Ellis, 1990). In beginner TRs, the intensive practice of decoding might enhance cumulative rehearsal (a strategy used in verbal memory), which in turn might lead to better sequential order memory performance. Additionally, the emergence of phonemic awareness and of orthographic representations might enhance the quality and precision of the language representations, which, in turn, would improve the encoding and retrieval of item information (Demoulin & Kolinsky, 2016). It is possible that children with RDs in the current study might be using strategies of beginning readers. Hence, better reading ability influenced better working memory predicted by FA in the left SLF. In contrast, TRs in the current study did not show this mediating effect of reading on the relationship between FA in the left SLF and working memory ability. The mediation analysis was also conducted while using SLF, working memory, and reading in other directions to test whether working memory mediated the relationship between FA in the SLF and reading ability, but no significant results were found. Hence, we can conclude that the only meaningful relations between the variables is when reading ability is a mediator for the relationship between FA in the left SLF and working memory ability.

### Limitations

Our results should be considered with the following limitation. Even though AFQ provides a method to assess variance within tracts, using the tensors model in AFQ entails discarding the small branches in the tract and crossing fibers in a voxel. Future studies utilizing more sophisticated methods for diffusivity analysis algorithms such as neurite orientation dispersion and density imaging (NORDI) (Barritt et al., 2018), as well as CHARMED, AxCaliber, or ActiveAx (Assaf & Alexander, 2014) are warranted.

### Conclusions

In summary, our results show the localized white matter tract differences between children with RDs and TRs overall and in relation to EFs and reading. These findings provide structural support to the involvement of EFs and especially of working memory, switching/inhibition in the extended Simple View of Reading model, and specifically in relation to single-word reading.

## ACKNOWLEDGMENTS

The authors would like to thank the families participating in the current study.

## AUTHOR CONTRIBUTIONS

Rola Farah: Formal analysis; Visualization; Writing – original draft; Writing – review & editing. Noam Glukhovsky: Writing – original draft; Writing – review & editing. Keri Rosch: Writing – review & editing. Tzipi Horowitz-Kraus: Conceptualization; Data curation; Funding acquisition; Methodology; Project administration; Resources; Supervision; Writing – review & editing.

## FUNDING INFORMATION

Tzipi Horowitz-Kraus, National Institute of Child Health and Human Development (https://dx.doi.org/10.13039/100000071), Award ID: HD086011.

## TECHNICAL TERMS

Diffusion tensor imaging (DTI): An advanced, noninvasive magnetic resonance imaging modality focused on the study of water diffusion in white matter fibers; provides information about the microstructure of the fibers, including orientation, degree of myelination and axonal density.
Fractional anisotropy (FA): Fractional anisotropy is a common measure often used in diffusion imaging and often used to quantify white matter integrity; is thought to reflect fiber density, axonal diameter, and fiber myelination with values that range from 0 (highly isotropic—poor white matter integrity) to 1 (highly anisotropic—good white matter integrity).
Automatic fiber quantification (AFQ): A popular software that identifies, automatically, major fiber tracts in the brain and quantifies tissue properties at multiple locations along the tract length, hence creating a Tract Profile.
Permutation-based multiple comparisons correction: A nonparametric permutation approach that provides a flexible approach to account for the multiple comparisons problem implicit in the standard neuroimaging data analysis by utilizing a locally pooled (smoothed) variance estimate.
Fisher *z*-transformation: The Fisher *z*-transformation is a formula to transform the sampling distribution of Pearson’s *r* (i.e., the correlation coefficient) so that it becomes normally distributed and can be used to calculate a confidence interval for Pearson’s correlation coefficient.
Moderated mediation analysis: The process of uncovering the relationship between a dependent (*X*) and independent (*Y*) variable that is transmitted through a mediator (*M*) variable and is conditional on values of a moderating variable (*W*).
